# Spirulina platensis extract addition to semen extender enhances cryotolerance and fertilizing potentials of buffalo bull spermatozoa

**DOI:** 10.1590/1984-3143-AR2020-0520

**Published:** 2021-08-02

**Authors:** Magdy Badr, Zaher Rawash, Ahmed Azab, Ragab Dohreg, Taha Ghattas, Mohamed Fathi

**Affiliations:** 1 Artificial Insemination and Embryo Transfer Department, Animal Reproduction Research Institute, Giza, Egypt; 2 Pathology Department, Animal Reproduction Research Institute, Giza, Egypt; 3 Biology of Reproduction Department, Animal Reproduction Research Institute, Giza, Egypt; 4 Department of Theriogenology, Faculty of Veterinary Medicine, Cairo University, Giza, Egypt

**Keywords:** spirulina, buffalo bull, spermatozoa, cryopreservation, lipid peroxidation

## Abstract

The objective of this study was to investigate the effect of Spirulina platensis extract (SPE) addition to the freezing extender on freezability, lipid peroxidation, ultrastructure alterations and fertilizing potentials of frozen-thawed buffalo bull spermatozoa. Semen samples were collected with artificial vagina from five adult fertile bulls and diluted with Tris-base extender containing SPE (1, 5, 10 and 20 μg/mL) or without SPE (control). Diluted semen was cooled to 4 °C throughout one hour and frozen in 0.25 mL straws: prior to being stored in liquid nitrogen. Cryopresreved spermatozoa were assessed for post-thawing sperm motility, viability, acrosomal integrity, ultrastructure changes, antioxidant activities, lipid peroxidation and fertility rate. The current results clearly indicated that adding 10μg/mL SPE to the freezing extender significantly improved (P< 0.05) post-thawing motility and decrease the percentage of acrosomal damage (51.67±6.02% and 16.33±1.46%, respectively) compared with the control (28.33±4.41% and 26.33±1.77%, respectively). Moreover, addition of 10 μg/mL SPE to the semen extender significantly diminished (P< 0.05) MDA concentration (10.66±2.40 nmol/10^9^) compared with the control (22.66±4.26 nmol/10^9^). Therefore, the present results revealed that addition of 10μgl/mL SPE to the freezing extender might improve semen quality and reduce cryodamage of the buffalo bull spermatozoa.

## Introduction

Semen cryopreservation offers many benefits to the livestock productiveness through dissemination of valuable genetic material by means of artificial insemination (Bucak et al., 2009). Achievement of any successful AI program depends on the proper management of semen collection, storage and handling (Leboeuf et al., 2000). Various conception rates were recorded in buffaloes; 33.3% during breeding season (Paul and Prakash, 2005) and 28.2% out of the breeding season (Baruselli et al., 2007). Although, many protocols have been developed for semen cryopreservation, sperm cryo-survival rates were still not optimum in buffalo. Cryopreservation induces some irreversible damages in sperm cells (Medeiros et al.*,* 2002). One of the most vital factors responsible for these damages is the unregulated increase of reactive oxygen species (ROS), which can lead to oxidative stress (Vemo et al., 2018). Oxidative stress (OS), occurs mainly as result of imbalance between the reactive oxygen species (ROS) and antioxidants capacity (El-Tohamy and El-Nattat, 2010). Oxidative stress induces damage to sperm by increasing lipid peroxidation rate and altering membrane function manifested with impaired metabolism, morphology, mobility and fertilizing potential, ROS is usually derived from three sources; mitochondria of sperm cells, plasma membrane and L- amino acid oxidases activity (Aitken, 2017). Balance between ROS production and their detoxification is an important factor for sperm survival and function before, during and after cryopreservation. Moreover, ROS are associated with lipid peroxidation, DNA and protein damages (Farber et al., 1990; Calamera et al., 2001; Neild et al., 2003), affecting the final usefulness of the cryopreserved semen. Lipid peroxidation leads to decreasing life span of spermatozoa either in vivo or after artificial insemination (Alvarez and Storey, 1982). Antioxidant molecules could not only reduce the impact of OS but also controlling physiological sperm functions (Aitken et al., 2004; Ford, 2004).

In order to counteract the deleterious effects of ROS, plants with antioxidant properties such as green tea extracts (Venkatachalam et al., 2012; Abshenas et al., 2011) and ethanolic extracts of Bersama engleriana (Vemo et al., 2017) had been used.

Spirulina is a planktonic blue green algae that considered as a traditional food of some Mexican and African people due to its enrichment with many valuable nutrients (Khan et al., 2005). Spirulina is considered as a strong natural antioxidant with anti-lipid peroxidation activity (Kurd and Samavati, 2015). In addition, Spirulina contains both enzymatic (superoxide dismutase, glutathione peroxidase, catalase and ascorbate peroxidase) and non-enzymatic (carotenoids, ascorbic acid, tocopherols and chlorophy-ll derivatives) as antioxidant protection system (Abd El-Baky et al., 2007). The positive effect of Spirulina was previously reported in swine sperm quality in terms of concentration, motility and post-storage viability (Granaci, 2007a). Also, Mizera et al., 2019 concluded that addition of spirulina to semen extender improves the bovine semen quality. In the same way Kistanova et al. (2009) reported that supplementation of diet with spirulina extract improves boar semen characteristics.

In rats, Nah et al. (2012) demonstrated that spirulina extracts had both antioxidant activity and anti-inflamatory action, in rabbits (El-Ratel and Gabr, 2020; Fouda and Ismail, 2017) concluded that addition of spirulina could improve rabbit semen production and its antioxidant potential. C-phycocyanin (C-PC) is considered an extracted biocompound from Arthrospira maxima with an antioxidant property, its addition to swine semen extender resulted in an increase of progressive motility and decrease of the intracellular ROS production (Antonio Marcos et al., 2018).

Despite all these, studies related to the effects of Spirulina on male reproduction were still limited. So, the current study aimed to investigate the effect of supplementation of buffalo bull semen extender with the hydro-ethanolic extract of Spirulina on the freezability, biochemical characteristics and fertilizing potentials of buffalo bull spermatozoa.

## Material and methods

### Spirulina Platensis Extract (SPE)

Spirulina was obtained from national research institute, algae biotechnology department, Egypt.

Dried Spirulina was extracted by soxhletation. The Spirulina powder (20g) was soaked in 1L of ultrapure water and shaken continuously for 24 h at room temperature. The mixture was then centrifuged at 5000 g for 10 min and the supernatant was filtered to remove the cell debris. The sample was then freeze-dried and the dried extract was stored at 4 °C till use (Chu et al., 2010).

### Extender preparation

All reagents and chemicals were obtained from Sigma-Aldrich.

The cryoprotective extender was Tris-based extender (375 mM Tris; 124 mM citric acid; 41.6mM glucose, 20% (v/v) egg yolk, 7% (v/v) glycerol, 25 mg gentamicin, and 50,000 IU penicillin; and dissolved in 100 mL deionized water, pH=6.8), Spirulina platensis extract (SPE) was added to the extender with different concentrations; 1,5,10 and 20 µg/mL or extender SPE free (control).

### Semen collection

Semen samples were collected from five healthy buffalo bulls aging 3-4 years and housed individually in pens at Animal Reproduction Research Institute farm (Cairo, Egypt). Two consecutive ejaculates were collected from each bull weekly for six weeks using an artificial vagina. The ejaculates were pooled to eliminate variability between the collected samples. Semen samples were assessed for volume, sperm concentration, percentage of motile spermatozoa and sperm morphology. The ejaculates with at least 70% progressive forward motility and 85% normal sperm morphology were used. All experiments were done with at least 3 replicates for each group. Animal care standards were followed and licensed by Animal Use Ethical Committee at Reproduction Research Institute (protocol number: 796-3-1-2020).

### Semen processing

After evaluation, the fresh semen samples were pooled and then split into five equal fractions and diluted at 30 °C with Tris-based extender alone (control) or supplemented with different concentrations of Spirulina extract (1, 5, 10 and 20 μg/mL). The fresh semen samples were transferred to pre warmed tubes. Diluted semen was cooled from 37 °C to 5 °C throughout 60 min in a cold cabinet. The cooled semen was loaded into 0.25 mL polyvinyl chloride straws (IMV, L'Aigle, France), horizontally placed in a refrigerator and kept at 4 °C for 1 h for equilibration. Straws were then placed 6 cm above the liquid nitrogen surface where the temperature was approximately −120 °C. After 15 min, straws were immersed directly into liquid nitrogen (−196 °C) for storage. The straws were stored at least for 24 hour before evaluation. Straws were thawed in water bath at 37 °C for 30 sec.

### Assessment of post-thawing sperm motility

Post-thawing sperm motility was assessed according to (Badr et al., 2010). Inbrief, The percentage of linear motile sperm was examined visually; three straws were thawed separately by immersion in a water bath at 37 °C for 30 sec, samples were placed on glass slides covered with a cover and then estimated at 37 °C by phase contrast microscope equipped with a warm stage at 200× magnification. Sperm motility estimation was performed in 3 different microscopic fields and the mean of them was recorded as the final motility score.

### Assessment of acrosomal integrity

Acrosomal integrity was assessed following thawing using silver nitrate stain. Sperm suspension was spread over slides and dried at room temperature. The preparations were fixed in ethyl alcohol 70% for 2 min then in ethyl alcohol 95% for another 2 min. The preparations were stained with the silver nitrate solution (100 mg**/**200 mL distilled water) for 2 h in an incubator at 65 °C. After the preparations were turned gold in color, the chemical reaction was interrupted and the preparations were rinsed several times with distilled water. The preparations were dried at room temperature. The dried preparations were analyzed for acrosomal integrity using the Olympus BX50 light microscope with a 100-fold magnification. At least 300 sperm cells were counted per slide and the percent of acrosome intact spermatozoa was calculated (El-Amrawi, 1992; El-Shahat et al., 2020).

### Ultrastructure analysis of the cryopreserved spermatozoa

The ultrastructure changes of the cryopreserved spermatozoa were evaluated by transmission electron microscopy (TEM). Five straws from each treatment were washed three times by centrifugation at 1000 g for 5 min using phosphate buffer saline (PBS). The frozen-thawed sample was prefixed for 2-3 h with PBS containing 2% glutaraldehyde, washed three times by centrifugation at 1000 g with PBS (pH 7.4) for 5 min at 4 °C and post-fixed in 1% osmium tetroxide for 1-2 h at 4 °C (Boonkusol et al., 2010). Spermatozoa were dehydrated in propylene oxide and embedded in epon resin. Ultrathin sections were cut using the Leica EM UC6 ultra microtome and stained with uranyl acetate and lead citrate. Randomly fields were examined by a transmission electronic microscope (JEOL-EM-100 S at TEM lab FA-CURP, Faculty of Agriculture, Cairo University -Research Park CURP). Images were captured by CCD camera model AMT, optronics camera with 1632 x 1632 pixel format as side mount configuration.

### Biochemical assays of superoxide dismutase (SOD), Glutathione reductase (GSH), Malondialdehyde (MDA) and Total Antioxidant Capacity (TAC)

SOD activity of frozen-thawed samples was measured at 560 nm on a spectrophotometer and expressed as units per milliliter according to Flohe and Otting (1984). Briefly, the increase in absorbance at 560 nm for 5 mint for control and for sample at 25 °C was measured. SOD activity (U/mL)= % inhibition×3.75. the assay is depending on the ability of SOD to prevent the reduction of nitroblue tetrazoliumdye.

GSH content was measured at 412 nm on a spectrophotometer and the values of GSH were expressed as units (Sedlak and Lindsay, 1968). Glutathione reductase activity (U/mL)= 4019× 340 nm/min.

The concentrations of Malondialdehyde (MDA), as indices of the lipid peroxidation in the semen samples were measured using the thiobarbituric acid reaction according to the method of Placer et al. (1966). In brief, an aliquot of semen sample (500 µL) was centrifuged at 800 xg for ten minutes, sperm pellets were gathered and resuspended in phosphate buffer saline then recentrifuged again, after centrifugation, 1 mL of deionized water was added to sperm followed by snap-freezing and store at -70 until usage. Malondialdehyde (nmol/10^9^)= A Sample/A Standard ×10

TAC content was measured at 505 nm on a spectrophotometer and the values of TAC were expressed as mM**/**L according to Kumar et al. (2017). Determination of tac was accomplished by the reaction of antioxidants of the sample with a definite amount of exogenouly added hydrogen peroxide. Tac (mM**/**L) = absorbance of blank – absorbance of sample ×3.33

### Artificial insemination of the synchronized buffaloes

Estrus was synchronized in normal cyclic buffaloes (n= 62) as mentioned by Hoque et al. (2011). In brief, at day (0); 5 mL buserelin; a synthetic analogue of GnRH (Receptal)^R^/ buffalo was injected, at day (7); 2 mL cloprostenol; a synthetic analogue of PGF2a (Estrumate)^R^ /buffalo was injected, at day (9); 5 mL receptal/ buffalo was injected, at day (10); artificial insemination was done by recto-cervical technique using frozen-thawed straw with 15 x 10^6^ spermatozoa. Insemination was done twice (AM and PM) for each buffalo, insemination was done in winter season, only cryopreserved semen with 10 µg/mL SPE and the control were used for AI trials, pregnancy was checked at day 35 using 7.5-10 MHz - linear-array transducer attached with ultrasound machine (Sonoscape A5, China).

### Statistical analysis

Experiments were repeated three times. data were analyzed by using Costat Computer Program, Version 3.03 copyright Cottort Software, and were compared by the least significant difference least (LSD) at 5% level of probability. The results were expressed as mean ±SEM. The mean values of the percentages of motile sperm, acrosome-intact sperm and enzymes activity were compared using Duncan’s multiple range test by one-way ANOVA procedure . Conception rates were compared using Student t-test, F-value was significant at (P < 0.05).

## Results

### Effect of SPE addition to semen extender on semen freezability

**Figure 1:**
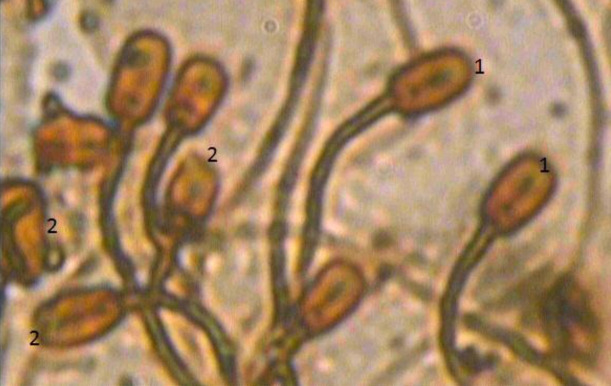
Silver nitrate staining of sperm acrosomes with magnification of (× 100), 1 refers to normal acrosome while 2 refers to damaged acrosome.

The results presented in Table 1 revealed that, addition of SPE to the freezing extender improved the freezability of buffalo bull spermatozoa compared with the control in dose dependent manner. The results obtained in the sample supplemented with 10 µg/mL of SPE were the highest of all tested samples and differed significantly compared with the control group. Addition of 10 µg/mL SPE significantly (P<0.05) improved post-thawing sperm motility and decreased the percentage of damaged acrosome (51.67±6.02% and 16.33±1.46%, respectively) compared with the control (28.33±4.41% and 26.33±1.77%, respectively) and 20 µg/mL (30.00±5.01% and 29.33±3.18%, respectively) (Figure 1).

**Table 1:** Effect of Spirulina platensis extract (SPE) addition to semen extender on buffalo bull semen freezability.

**Treatment**	**Post-thawing motility (%)**	**Acrosomol abnormality (%)**
**Control**	28.33±4.41 ^c^	26.33±1.77 ^ab^
**SPE 1 µg/mL**	36.67±4.40 ^abc^	19.00±2.09 ^bc^
**SPE 5 µg/mL**	45.00±2.89^ab^	20.33±2.91 ^bc^
**SPE 10 µg/mL**	51.67±6.02 ^a^	16.33±1.46 ^c^
**SPE 20 µg/mL**	30.00±5.01 ^bc^	29.33±3.18 ^a^

Values with different superscripts within the same columns differed significantly at (P<0.05).

### Effect of SPE addition to semen extender on antioxidant activity of the cryopreserved semen and lipid peroxidation

Data regarding the effect of SPE addition to the freezing extender on the total antioxidant capacity (TAC), SOD, GSH activities and lipid peroxidation of the cryopreserved buffalo bull spermatozoa were presented in Table 2. Supplementation of freezing extender with 10 µg/mL SPE significantly (P<0.05) increased TAC, SOD and GSH concentrations (0.49±0.014, 58.33±1.76 and 61.66±2.02) compared with the control (0.30±0.049, 33.66±3.29 and 40.66±1.46, respectively). Whereas, the present data demonstrated that, in vitro provision of semen extender with 10 µg/mL SPE significantly (P<0.05) diminished lipid peroxidation of the frozen-thawed semen (10.66±2.40 nmol/10^9^) compared with the control (22.66±4.26 nmol/10^9^).

**Table 2:** Effect of SPE addition to semen extender on anti-oxidant activities and lipid peroxidation of cryopreserved buffalo bull spermatozoa.

**Treatment**	**TAC**	**SOD**	**GSH**	**Lipid peroxidation**
	**(mM/L)**	**(U/mL)**	**(U/mL)**	**(nmol/10^9^)**
**Control**	0.30±0.049^b^	33.66±3.29 ^c^	40.66±1.46^c^	22.66±4.26^a^
**SPE 1 µg/mL**	0.38±0.042^ab^	43.66±4.91 ^bc^	51.33±4.98 ^abc^	16.33±2.03^ab^
**SPE 5 µg/mL**	0.40±0.052^ab^	51.33±4.34 ^ab^	56.00±4.59^ab^	14.33±2.61^ab^
**SPE 10 µg/mL**	0.49±0.014^a^	58.33±1.76^a^	61.66±2.02^a^	10.66±2.40^b^
**SPE 20 µg/mL**	0.32±0.018^b^	38.66±5.05 ^bc^	47.00±2.65^bc^	24.00±4.05^a^

Values with different superscripts in the same columns were significantly different at (P<0.05). TAC: total antioxidant capacity; SOD: superoxide dismutase; GSH: glutathione reductase.

### Effect of SPE addition to semen extender on fine structure of the cryopreserved spermatozoa

**Figure 2:**
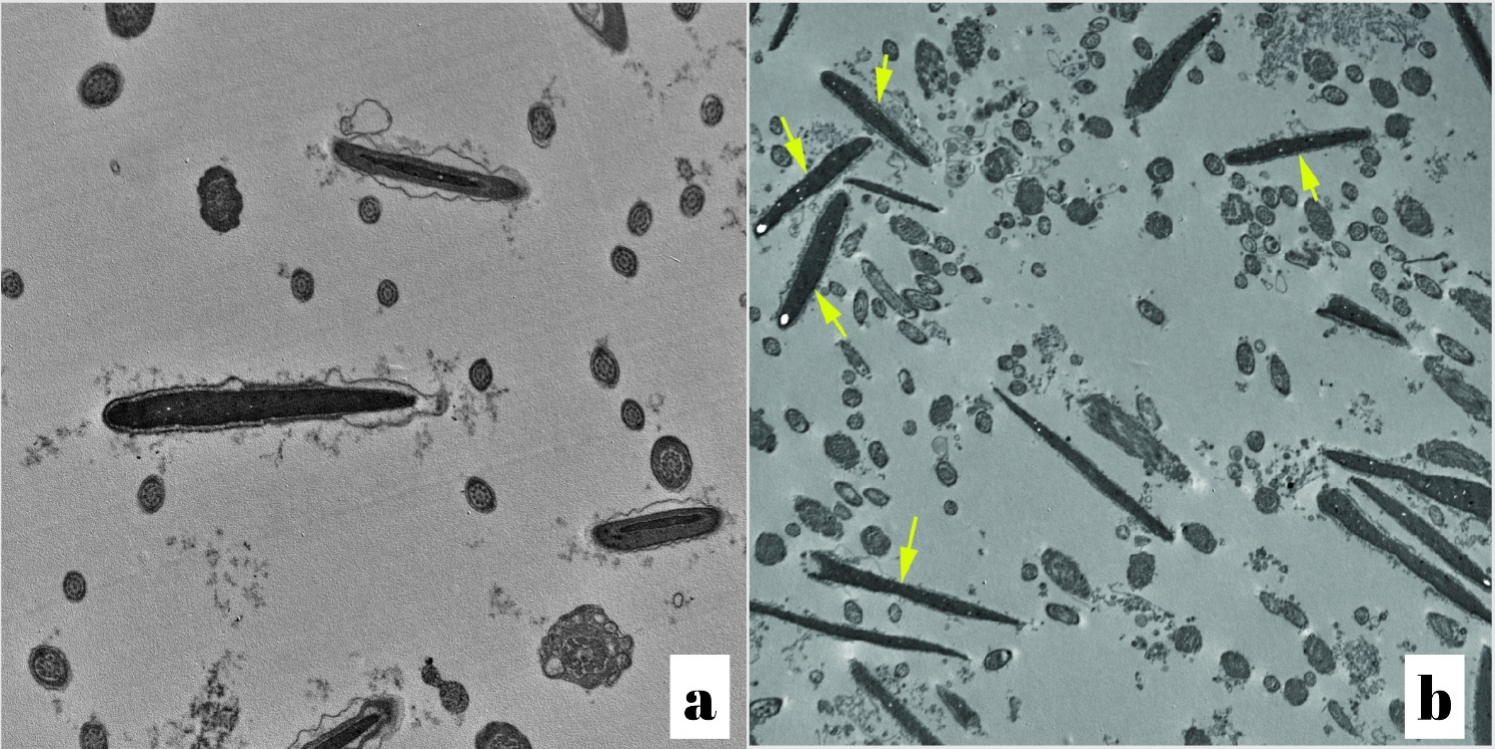
(a) Electron micrograph for a sagital section in the sperm head from a frozen-thawed semen sample of control group showing swollen plasma membrane (PM) (× 15000); (b) Electron micrograph for a sagital section in the sperm head from frozen-thawed semen sample treated with spirulina illustrating intact plasma membrane (PM) and the outer and inner acrosomal membranes are intact and the subacrosomal space is evident (× 15000), as referred by yellow arrows.

**Figure 3:**
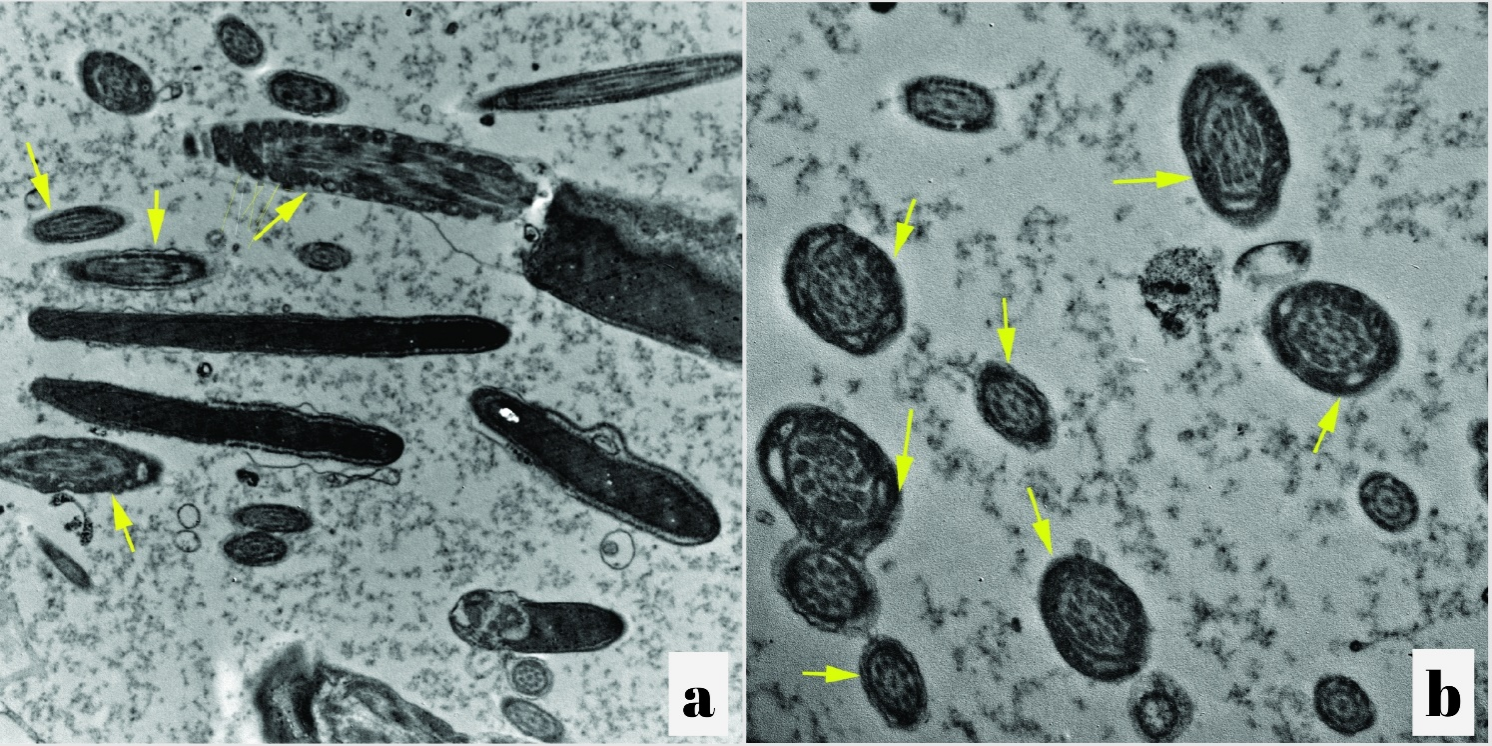
(a) Electron micrograph of a cross section in the neck region (note the presence of mitochondria in different orientation) of sperm from a frozen-thawed semen sample of control group showing severe degeneration (marked vacuolation) in the mitochondria that contained electron-translucent spaces with complete absence of the transverse cristae and some mitochondria are completely disappeared (× 30000) as referred by yellow arrows; (b) Electron micrograph of a cross section in the mid-piece region of the tail from a frozen-thawed semen sample treated with spirulina illustrating good mitochondrial dense electron spaces with appeared transverse cristae (× 30000) as referred by yellow arrows.

Frozen-thawed spermatozoa in the control group showed, swollen plasma membrane segmentation of the outer acrosomal membrane and swollen acrosome, moreover, severe degeneration and marked vacuolation in the mitochondria with complete absence of the transverse cristae (Figure 2a and 2b). Electron microscopic images of sagital sections of the frozen thawed sperm cells treated with 10 µg/mL SPE illustrated a well defined and intact plasma membrane, intact acrosomal membranes, homogenous mitochondria and high-quality mitochondrial dense electron spaces with appeared transverse cristae (Figure 3aand3b).

### Effect of SPE addition to semen extender on fertility following post-thawing artificial insemination

As shown in Table 3, the conception rate was significantly (P<0.05) higher following artificial insemination of the synchronized buffaloes with frozen-thawed semen samples supplemented with 10µg/mL SPE (62.50±1.72%) compared with the control (26.67±1.26%).

**Table 3:** Effect of SPE addition to semen extender on conception rate following artificial insemination.

**Treatment**	**No of buffalo**	**No of conceived buffalo**	**Conception rate**
**control**	30	8	26.67±1.26 ^b^
**SPE 10 µg/mL**	32	20	62.50±1.72 ^a^

Values with different superscript in the same columns were significantly different at (P<0.05).

## Discussion

The goal of cryopreservation is to be able to preserve the quality of spermatozoa for long term, but there are several factors that could affect the post-thawing outcomes (Azevedo, 2006). One of the most important factors is the extender constituents. Supplementation of the freezing extender with SPE was significantly enhancing the characteristics of the cryopreserved buffalo spermatozoa, such as progressive motility, acrosome integrity, antioxidant activity, moreover reducing lipid peroxidation and preserve the fertilizing potential of the cryopreserved spermatozoa, in a dose dependent manner, while with increasing dose to 20 µg/mL, a decline in sperm quality may be observed. The limitation of this study was the small sample size.

The results were in line with the findings of (Kistanova et al., 2009; Nah et al., 2012; El-Tantawy, 2016; Djalil et al., 2019; Mizera et al., 2019). The previous authors reported the positive effects of SPE on motility, antioxidant activity and membrane integrity of mammalian spermatozoa. Moreover, (Granaci, 2007b; Mizera et al., 2019) reported that biological extract of Spirulina has a positive effect on sperm motility and viability after storage. In the same way of our results (Mizera et al., 2019) reported a significant incresea in motility percentage of bovine sperm from 49.36% in control group to 61.23% in spirulina treated group. Additionally, Spirulina supplementation also has an optimistic outcome on the process of spermatogenesis in rats (Nah et al., 2012; Esener et al., 2017).

Spirulina contains vital compounds, such as protein (50-70% on DM basis) with all essential amino acids (Farag et al., 2016), essential fatty acids, alpha-linolenic, gamma-linolenic and linoleic (Mendes et al., 2003), nutraceutical pigments (Keservani et al., 2015), vitamins as thiamine, nicotinamide, riboflavin, folic acid, pyridoxine, vitamins A, D and E (Hosseini et al., 2013) and minerals like Ca, K, Cr, Cu, Mn, Fe, P, Mg, Na, Zn and Se (Babadzhanov et al., 2004).

It is also known Spirulina has an antioxidant potential (El-Tantawy, 2016) and anti-diabetic effects as it plays a crucial role in decreasing blood glucose level, regulating cholesterol and improving insulin resistance (Gupta et al., 2010).

A variety of speculations have been proposed by several authors to explain the protective mechanism of SPE on mammalian spermatozoa. The beneficial effect of SPE may be due to its anti-inflammatory activity (Cingi et al., 2008). Rahman et al. (2012) reported that Spirulina may increase the production of both immunostimulatory and immuno-mediators. Moreover, many reports recorded the significant effect of Spirulina as free radicals scavenging. Our results had been confirmed the antioxidant activities of spirulina, moreover its crucial role in decreasing the lipid peroxidation. Another theory for the beneficial effects of SPE on the vital parameters of the cryopresreved spermatozoa may be attributed in fact to its antioxidant activities, scavenge ROS and protect sperm cell from toxic oxygen metabolites causing lipid peroxidation of sperm plasma membrane during cryopreservation (Bilodeau et al., 2001). Antonio Marcos et al. (2018) reported that the addition of C-phycocyanin as an extracted compound from Arthrospira maxima in the boar semen extender could control the production of intracellular ROS production that couldy positively reflect on the increase in the percentage of progressive motility.

Membrane of mammalian spermatozoa is sensitive to oxygen-induced damage; excessive formation of either ROS or Reactive Nitrogen Species (RNS) by abnormal spermatozoa has been identified as one of the few definite reasons for male infertility. Oxidative stress is a condition associated with an increased rate of cellular damage induced by oxygen and oxygen-derived oxidants (Sikka et al., 1995), that have been implicated in impaired semen quality (Joyce, 1987). Mammalian spermatozoa are susceptible to lipid peroxidation which destroys the structure of the lipid matrix of plasma membrane, due to the attacks of ROS formed from reduction of oxygen during cryopreservation. The damage of lipid matrix finally causes irreversible damage to the plasma membrane, decreased sperm motility, loss of fertility and damage of the sperm DNA (Storey, 2008). The current data confirms this theory as SPE supplementation increased the activity of antioxidant enzymes and reduced lipid peroxidation of the cryopreserved spermatozoa compared with the control. Similar antioxidant potential of Spirulina has been observed (Gutiérrez-Rebolledo et al., 2015; Asghari et al., 2016; Bashandy et al., 2016).

The present results revealed a significant higher conception rate in the artificially inseminated buffaloes with frozen-thawed semen supplemented with 10 µg/mL SPE, this may be attributed to the valuable effects of SPE on acrosomal integrity, decrease lipid peroxidation, enhancing the membrane integrity and antioxidant activities of the cryopreserved spermatozoa that would eventually enhance the fertilizing potentials of the cryopresreved spermatozoa.

Results of the current study confirmed that increasing of the used SPE to 20 µg/mL was detrimental to the cryopreserved buffalo bull spermatozoa. In agreement with this finding (Mizera et al., 2019) reported that, high concentrations of SPE could lead to a significant reduction in sperm motility and viability.

## Conclusion

The emerged results of this study clearly demonstrate that supplementation of semen extender with SPE exerts valuable effects on the quality and the fertilizing potentials of the cryopreserved buffalo spermatozoa in a dose dependent manner. 10 µg/mL SPE addition appeared to be the best concentration. These beneficial effects appeared due to the improvement of the antioxidant activities and the diminishing rates of lipid peroxidation of the cryopreserved spermatozoa.
